# Efficient hydrogen evolution in transition metal dichalcogenides via a simple one-step hydrazine reaction

**DOI:** 10.1038/ncomms11857

**Published:** 2016-06-10

**Authors:** Dustin R. Cummins, Ulises Martinez, Andriy Sherehiy, Rajesh Kappera, Alejandro Martinez-Garcia, Roland K. Schulze, Jacek Jasinski, Jing Zhang, Ram K. Gupta, Jun Lou, Manish Chhowalla, Gamini Sumanasekera, Aditya D. Mohite, Mahendra K. Sunkara, Gautam Gupta

**Affiliations:** 1 Materials Physics and Applications (MPA-11), Los Alamos National Laboratory, Los Alamos, New Mexico 87545, USA; 2 Chemical Engineering and Conn Center for Renewable Energy Research, University of Louisville, Louisville, Kentucky 40292, USA; 3 Materials Science and Engineering, Rutgers University, Piscataway, New Jersey 08854, USA; 4 Materials Science and Technology (MST-6), Los Alamos National Laboratory, Los Alamos, New Mexico 87545, USA; 5 Materials Science and NanoEngineering, Rice University, Houston, Texas 77005, USA; 6 Chemistry, Pittsburg State University, Pittsburg, Kansas 66762, USA

## Abstract

Hydrogen evolution reaction is catalysed efficiently with precious metals, such as platinum; however, transition metal dichalcogenides have recently emerged as a promising class of materials for electrocatalysis, but these materials still have low activity and durability when compared with precious metals. Here we report a simple one-step scalable approach, where MoO_x_/MoS_2_ core-shell nanowires and molybdenum disulfide sheets are exposed to dilute aqueous hydrazine at room temperature, which results in marked improvement in electrocatalytic performance. The nanowires exhibit ∼100 mV improvement in overpotential following exposure to dilute hydrazine, while also showing a 10-fold increase in current density and a significant change in Tafel slope. *In situ* electrical, gate-dependent measurements and spectroscopic investigations reveal that hydrazine acts as an electron dopant in molybdenum disulfide, increasing its conductivity, while also reducing the MoO_x_ core in the core-shell nanowires, which leads to improved electrocatalytic performance.

## Abstract (web summary)

Transition metal dichalcogenides are promising hydrogen evolution catalysts however they can require expensive processing steps to enhance their activity. Here, the authors report a one-step activation step in which room temperature hydrazine treatment results in much enhanced electrocatalytic performance.

## Graphical abstract

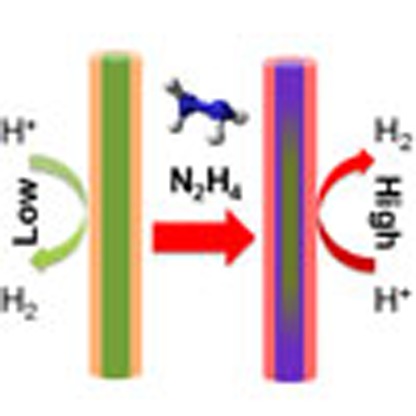

Hydrogen has the potential to be a zero-emission, renewable fuel; however, today it is primarily obtained from thermal steam reforming of natural gas^1^,^2^,^3^,^4^. It can be produced via water splitting, but the high cost of precious metal catalysts and rare earth materials that are currently used present a challenge to large scale implementation^5^,^6^,^7^,^8^. Recently, layered transition metal dichalcogenides (TMDs), such as WS_2_, MoS_2_ and so on, have been explored as a viable alternative to precious metal catalysts^9^,^10^. Bulk MoS_2_ powders have limited catalytic activity due to an inert crystal basal plane and low in-plane conductivity^11^. High electrocatalytic activity can be achieved by either increasing the exposure of the active edge planes^12^,^13^,^14^,^15^,^16^,^17^, increasing the conductivity of MoS_2_ (refs 18, 19, 20), phase transformation^21^,^22^,^23^,^24^,^25^,^26^, use of a co-catalyst^27^ or a combination of these approaches^28^,^29^,^30^. Of these techniques, phase transformation from semiconducting (bulk hexagonal 2H-MoS_2_) to a metastable trigonal crystal structure (1T-MoS_2_), which has metallic properties and does not suffer from anisotropy, has recently shown the most promise for electrochemical and opto-electronic applications^28^,^31^,^32^,^33^. Phase-transformed TMDs, obtained via lithium intercalation, result in very efficient hydrogen evolution reaction (HER) catalytic characteristics; however, the processing conditions are expensive, time consuming (days), involve use of inert glove box atmosphere and often require elevated temperatures (100 °C). Lithium processing has also been shown to result in the formation of Li_2_S nanoparticle contaminants^9^. High-aspect ratio structures, such as one-dimensional core-shell nanowires^29^, also have the potential to achieve high HER activity. Although these structures possess properties such as high surface area and a conductive reduced oxide core, the primary drawback is that synthesis methods lead to the relatively inert basal plane of the MoS_2_ shell growing parallel to the length of the nanowire. This reduces the exposure of available active edge sites, resulting in lower catalytic activity for HER than theoretically achievable.

Hydrazine has been well researched as a reducing agent in two-dimensional (2D) reduced graphene oxide^34^ and demonstrated as an n-type dopant in graphene^35^,^36^, single-walled carbon nanotubes, both semiconducting and metallic^37^,^38^, as well as observed in inorganic nanocrystalline systems^39^,^40^, but its effects on layered TMDs and electrocatalytic properties have not been previously investigated to date. Chemical modification of the MoS_2_ inert basal plane to increase its charge carrier concentration, that is, electron doping, could lead to marked improvement in electrocatalytic activity.

Here we report a simple process, in which exposure of the MoO_x_/MoS_2_ core-shell nanowire arrays, as well as pure MoS_2_ particles and 2D sheets, to dilute hydrazine (N_2_H_4_) results in a marked improvement in catalytic activity towards HER, that is, both a significant improvement in overpotential (∼100 mV versus the reversible hydrogen electrode (RHE)), which is among the lowest reported HER overpotentials for any MoS_2_ architecture, and a 10-fold increase in current density (∼2 to 22 mA cm^−2^). Detailed characterization and conductivity measurements of MoO_x_/MoS_2_ core-shell nanowires, as well as pure MoS_2_ particles and sheets, show that hydrazine is acting as an electron dopant, donating electrons to increase the conductivity of MoS_2_, which leads to improved electrocatalytic activity. In the case of the core-shell nanowires, hydrazine further reduces the oxide core, which enhances conductivity and facilitates the charge transfer kinetics in the system, synergistically improving the HER performance of core-shell nanowires after exposure to hydrazine. This ‘activation' of the normally inert TMD basal plane by electron doping from hydrazine presents a unique opportunity to serves as a novel direction for efficient catalytic development and the use of simple processing techniques that can rival state-of-art platinum catalysts.

## Results

### Synthesis and characterization of MoO_x_/MoS_2_ architectures

**Figure 1:**
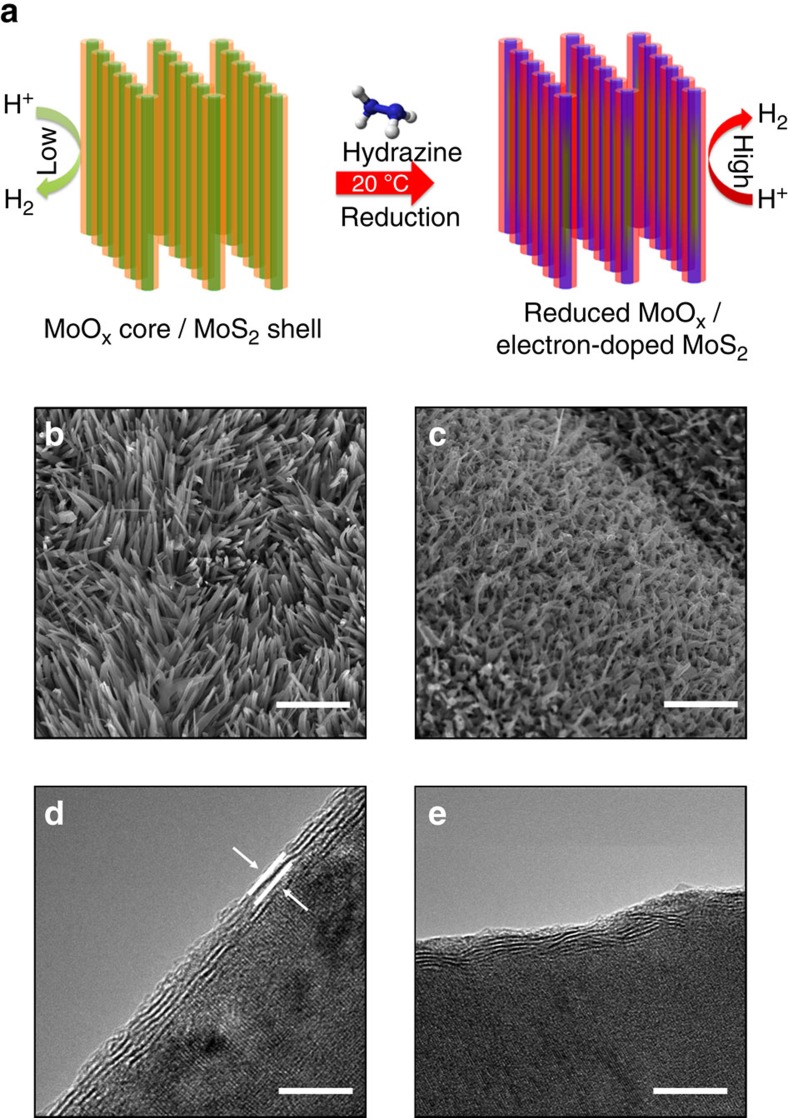
Schematic representation of hydrazine treatment and microscopic analysis of MoO_x_/MoS_2_ core-shell nanowires. (**a**) Schematic representation of exposure of MoO_x_/MoS_2_ core-shell nanowire array to dilute hydrazine under ambient conditions. (**b**) SEM image of as-grown MoO_x_/MoS_2_ core-shell nanowire array. Scale bar, 2 μm. (**c**) SEM imaging MoO_x_/MoS_2_ core-shell nanowires following dilute hydrazine treatment, showing that the overall nanowire morphology is maintained. Scale bar, 2 μm. (**d**) High-resolution TEM (HRTEM) of as-grown nanowire, showing thin (∼3–5 nm) MoS_2_ shell on a single crystal MoO_x_ core. The MoS_2_ has the typical interlayer spacing of 6.2 Å, denoted in image by two parallel lines. Scale bar, 5 nm. (**e**) HRTEM of the MoO_x_/MoS_2_ core-shell nanowire after exposure to hydrazine. Scale bar, 5 nm.

Figure 1a shows a schematic representation of a simple hydrazine treatment; the as-grown MoO_x_/MoS_2_ core-shell nanowires are exposed to a dilute hydrazine (1% in water) at room temperature for 10 min. Figure 1b shows the scanning electron microscopic (SEM) image of an as-grown, vertically oriented MoO_x_/MoS_2_ core-shell nanowire array on SiO_2_ substrates. Nanowires are typically 1–2 μm in length, with diameters of 20–50 nm. A thin (2–5 nm) highly oriented crystalline MoS_2_ shell is grown epitaxially on a single-crystal reduced MoO_x_ core, as can be seen using high-resolution transmission electron microscopy (Fig. 1d). MoO_3_ nanowire arrays are deposited on SiO_2_ substrates using chemical vapor deposition (CVD), followed by the sulfurization reaction at 300 °C under low pressures of 99% H_2_S (100 mTorr) for 2 h, leading to a thin (2–5 nm) single crystalline MoS_2_ on a single crystal MoO_x_ core. The synthesis methods of the MoO_x_/MoS_2_ core-shell nanowires are described in more detail elsewhere^29^,^30^,^41^. After exposure to hydrazine, SEM (Fig. 1c) shows minimal disruption of the nanowire morphology and there is no evidence of crystallographic disruption of the MoS_2_ shell, as shown in Fig. 1e.

### Electrochemical performance of materials

**Figure 2:**
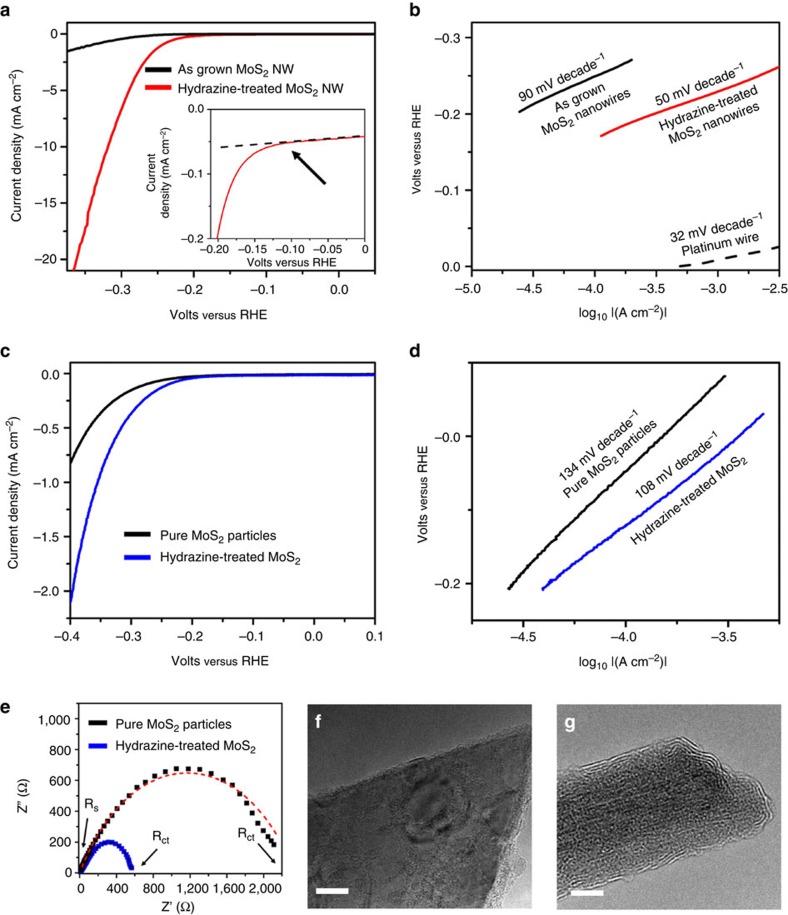
Electrochemical analysis of MoO_x_/MoS_2_ core-shell nanowires and MoS_2_ particles. (**a**) Linear sweep voltammetry for as-grown MoO_x_/MoS_2_ core-shell nanowire (black curve) and after exposure to 1% Hydrazine (red curve). (**b**) Tafel slopes for as-grown MoO_x_/MoS_2_ core-shell nanowires (black curve), 1% hydrazine-treated nanowires (red curve), and a platinum wire (dotted black curve). (**c**) Linear voltammograms and corresponding (**d**) Tafel slope analysis of MoS_2_ particles before (black curve) and following exposure to dilute hydrazine (blue curve). (**e**) Electrochemical impedance spectroscopy (EIS) Nyquist plots of the MoS_2_ particles following exposure to hydrazine, with line fits shown by dotted lines. (**f**) High-resolution TEM (HRTEM) of 2H-MoS_2_ particle. Scale bar, 5 nm. (**g**) HRTEM of as-grown MoO_x_/MoS_2_ core-shell nanowire. Scale bar, 10 nm.

The nanowires are dispersed in distilled water, removing any agglomerated hydrazine on the surface and transferred (∼70 μg cm^−2^) to a glassy carbon electrode for electrochemical characterization. Figure 2a shows a linear sweep voltammogram of the as-grown and hydrazine-treated nanowires. All of these electrocatalytic measurements are corrected for ohmic potential (iR) losses in the system (∼12 ohms); this resistance calculation is shown in Supplementary Fig. 1. The measured HER onset potential for the as-grown nanowires is approximately −200 mV versus RHE and a current density of ∼2 mA cm^−2^ at −0.35 V versus RHE is obtained. After exposure to 1% hydrazine, the onset potential improves to approximately −100 mV versus RHE and the current density increases to ∼22 mA cm^−2^ at −0.35 V. Hydrazine treatment on multiple nanowire samples indicate that this electrochemical performance is highly reproducible (Supplementary Fig. 2).

The rate of hydrogen evolution is limited by either proton adsorption onto an active site or evolution of the formed hydrogen from the surface. A high Tafel slope (120 mV per decade) is indicative of proton adsorption (Volmer step) as the rate-limiting step, while a lower Tafel slope (30 or 40 mV per decade) indicates that the evolution of molecular hydrogen from the catalyst is rate limiting (Heyrovsky or Tafel step, respectively)^7^,^42^,^43^. Figure 2b shows the Tafel plots for these voltammograms. The as-grown MoO_x_/MoS_2_ core-shell nanowires show a Tafel slope of 90 mV per decade, suggesting that adsorption of protons from the electrolyte is the rate-limiting step. Since the relatively inactive basal plane of the 2H-MoS_2_ shell is grown parallel to the MoO_x_ core nanowire, there are fewer catalytically active sites available for proton adsorption. After exposure to 1% hydrazine, the Tafel slope decreases to 50 mV per decade, indicating that the evolution of hydrogen via the combination of two adsorbed protons becomes the rate-limiting step. These results show that the hydrazine treatment significantly facilitates the adsorption of protons onto the catalyst surface. For comparison, the Tafel slope of a platinum wire is shown in Fig. 2b (dashed black curve); platinum has a Tafel slope of ∼30 mV per decade, which indicates proton adsorption is favourable and hydrogen evolution is the rate-limiting step^42^,^43^.

To corroborate the effects of hydrazine on TMDs without a core-shell nanowire architecture, pure MoS_2_ particles are exposed to dilute hydrazine. Figure 2c shows the linear voltammetry plots of bulk MoS_2_ powder with and without hydrazine. Bulk powder shows poor catalytic activity for the HER; following the hydrazine treatment, an improvement in both the current density and HER overpotential is observed. Tafel analysis (Fig. 2d) shows a decrease in the Tafel slope of the pure 2H-MoS_2_ particles, indicative of increased favourability for proton absorption onto the catalysis surface. Furthermore, electrochemical impedance spectroscopy of the MoS_2_ particles before and after hydrazine treatment (Fig. 2e) show a significant decrease in the charge transfer resistance. A R_s_-(CPE-R_ct_) circuit diagram is used to fit the experimental electrochemical impedance spectroscopy data. The solution resistance (*R*_s_) remains nearly constant (∼12 Ω), whereas the charge transfer resistance (*R*_ct_) decreases from ∼2,340 to ∼625 Ω, indicative of enhanced conductivity after the hydrazine treatment.

To quantify the improvement in catalytic activity following the hydrazine treatment, turnover frequency (TOF) is calculated for the MoO_x_/MoS_2_ core-shell nanowires and MoS_2_ particles using the following equation:


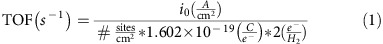


Exchange current densities (*i*_*0*_) are calculated from the Tafel equation, while the number of active sites is calculated from cyclic voltammograms of MoS_2_ particles and MoO_x_/MoS_2_ core-shell nanowires, both as-grown and treated with hydrazine. Following HER measurements, the potential applied to the working electrode was driven to high oxidation potentials (∼1.4 V versus RHE) to convert MoS_2_ particles to MoO_3_ (ref. 44). Calculation of active sites was obtained from the reduction charge transfer (MoO_3_ → Mo^0^) occurring at ∼ −0.3 to 0.0 V (ref. 29), assuming that each Mo^3+^ reduced to Mo^0^ corresponded to one MoS_2_ site. The results of these electrochemical decompositions to calculate surface area are shown in Supplementary Figs 3 and 4. The number of active sites for MoS_2_ bulk particles are similar before and after hydrazine treatment, 9.0 × 10^14^ and 9.8 × 10^14^ MoS_2_ sites per cm^2^, while Tafel analysis indicates an increase in exchange current density from 8.4 × 10^−6^ to 1.7 × 10^−5^ A cm^−2^. Using these values, the calculated TOF values increase for the MoS_2_ particles following the hydrazine treatment, from 0.03–0.05 s^−1^, while the number of active sites remain relatively unchanged. Summary of these obtained values, shown in Table 1, are consistent with reports by other researchers^31^,^45^,^46^.

A similar analysis process is performed to calculate TOF values for the MoO_x_/MoS_2_ core-shell nanowires. The −0.3 to 0.0 V region used in the particle calculations is more convoluted in the nanowires' case with the combination of core and shell oxidation–reduction peaks (Supplementary Fig. 4). Thus, the oxidation of MoS_2_ to MoO_3_ involving 11 e^−^ was used as the region for determining the number of MoS_2_ sites (as explained by Chen *et al*.)^29^. Interestingly, the series of oxidation–reduction decomposition peaks observed in the as-grown nanowires, resulting from the MoO_x_ core, are not observed following hydrazine treatment. This suggests that the hydrazine, in addition to doping of MoS_2_ shell, also reduces the oxide core, which increases its conductivity. The calculated number of MoS_2_ sites per surface area for the as-grown MoO_x_/MoS_2_ core-shell nanowires is ∼5.9 × 10^14^ sites per cm^2^. There is almost no change in the concentration of active sites in the nanowires following exposure to hydrazine, 6.0 × 10^14^ sites per cm^2^; this is confirmed by no observed physical change from SEM and TEM analysis (Fig. 1). Exchange current densities calculated from the obtained Tafel equations are 7.5 × 10^−6^ and 4.5 × 10^−5^ A cm^−2^ for the as-grown and hydrazine-treated MoO_x_/MoS_2_ core-shell nanowires, respectively. This results in an increase of TOF from 0.04 to 0.2 s^−1^. This fivefold improvement in TOF quantitatively shows the effect of hydrazine treatment on the electrocatalytic properties of MoS_2_ shelled nanowires.

Although, there is clear improvement in HER performance characteristics, the magnitude of the change in the bulk MoS_2_ is lower when compared with nanowires. This can be attributed to the difference in structure; the MoS_2_ powder particles are composed of several tens of molecular MoS_2_ layers with random orientations, as seen in Fig. 2f. This is in contrast to the core-shell nanowires, which have a few molecular layer thick MoS_2_ shell on an oxide core, as shown in Fig. 2g.

**Figure 3:**
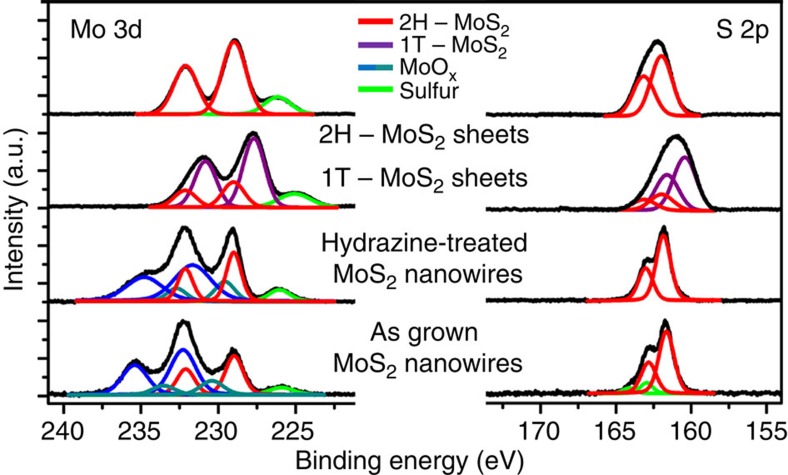
XPS Spectroscopy following hydrazine treatment. Spectra for MoO_x_/MoS_2_ core-shell nanowire array before and after hydrazine treatment, showing the Mo 3d, S 2s, and S 2p core level binding energies. The red curves denote the Mo 3d and S 2p signals corresponding to 2H-MoS_2_, with the purple curves showing the shift resulting from the phase transformation to 1T-MoS_2_. The MoO_x_ core seems to be a mixed phase valence, the Mo 3d oxidation states are shown by the dark and light blue curve, and sulfur signals are denoted by green.

Hydrazine is a reducing agent, as well as an electron dopant; we hypothesize that it interacts with the oxide core, further reducing it and increasing the intra-particle conductivity of the nanowire, while hydrazine also electron dopes the MoS_2_ surface. To test the hypothesis of reduction of oxide core, pure MoO_3_ nanowires are treated with dilute 1 % hydrazine, reported in the Supplementary Fig. 5. Pure MoO_3_ is catalytically inactive and decomposes quickly in acid solutions^47^ As expected, the MoO_3_ nanowires show no catalytic activity and decompose in the 0.5 M H_2_SO_4_. When exposed to hydrazine before testing, the MoO_3_ reduction and oxidation peaks are no longer observed, suggesting that the oxide has been reduced. This experiment supports a synergistic mechanism; hydrazine improves electron conductivity of the nanowire core, that is, intra-particle conductivity, while also electron doping the MoS_2_ surface (shown in pure MoS_2_ powder). The MoO_x_/MoS_2_ core-shell nanowire morphology, combined with the hydrazine treatment, uniquely provides architecture for an optimized electrocatalyst. The reduction of the MoO_x_ core by hydrazine is also confirmed by X-ray photoelectron spectroscopy (XPS) of a MoO_x_/MoS_2_ core-shell nanowire array in Fig. 3.

### Spectroscopic investigation of MoO_x_/MoS_2_ architectures

The core level binding energies of molybdenum and sulfur in as-grown MoO_x_/MoS_2_ core-shell nanowires are analysed using XPS, depicted in Fig. 3. The as-grown MoO_x_/MoS_2_ core-shell nanowires show strong doublet peaks at 229 eV, which are consistent with literature values for Mo^4+^ 3d_5/2_ and Mo^4+^ 3d_3/2_ of 2H-MoS_2_, shown by the red curves^21^. The MoO_x_ core results in a convolution of two oxidation states for Mo^6+^ in the reduced oxide core, shown by the dark and light blue signals at ∼232.6 and 230.6 eV; these binding energies suggest MoO_3_ and reduced MoO_3−*x*_. This is a result of incomplete reaction between the original MoO_3_ nanowires and the H_2_S during synthesis^29^. Following exposure to hydrazine, the partially reduced oxide core continues to reduce, indicated by the shift in Mo 3d signal towards lower binding energies of the further reduced oxide core (MoO_x_), ∼230.6 and 229.6 eV, respectively. It is established that reduced molybdenum oxide has almost metallic conductivity^48^; hydrazine, as a reducing agent, is increasing the intra-particle conductivity of the nanowire core. Despite the shift in Mo corresponding to the molybdenum oxide core, there is no shift in the MoS_2_ Mo 3d binding energies. When observing the S 2p signal for the MoS_2_ nanowire shell, the as-grown sample shows the clear doublet signal at ∼162 eV, typical for 2H-MoS_2_. After exposure to hydrazine, there is no detectable shift in the peak positions of the Mo 3d binding energies for 2H-MoS_2_ and only a slight broadening of the S 2p binding energy, but no noticeable shift. For comparison, the XPS spectra for chemically exfoliated 1T-MoS_2_ and 2H-MoS_2_ sheets are shown, modified from Cummins *et al*.^30^ It is well established that the phase transition from semiconducting 2H-MoS_2_ to metallic 1T-MoS_2_ has a corresponding large XPS binding energy shift of ∼ 0.9 eV (ref. 21), which is clearly evident in the chemically exfoliated control sample, but is not observed during the hydrazine treatment; it appears that hydrazine does not induce phase transition of MoS_2_ from 2H to 1T phase. This lack of phase transformation from 2H to 1T is also corroborated using Raman spectroscopy. The Raman analysis of MoS_2_ is discussed in Supplementary Note 1, with the Raman spectra shown in Supplementary Fig. 6. It has been shown that the 1T-MoS_2_ crystal phase results in unique Raman excitations, which can distinguish the metastable phase from the stable 2H-MoS_2_ structure^21^,^30^; these excitations are not detected in the hydrazine-treated samples.

### Electrical measurements on nanowires and 2D materials

**Figure 4:**
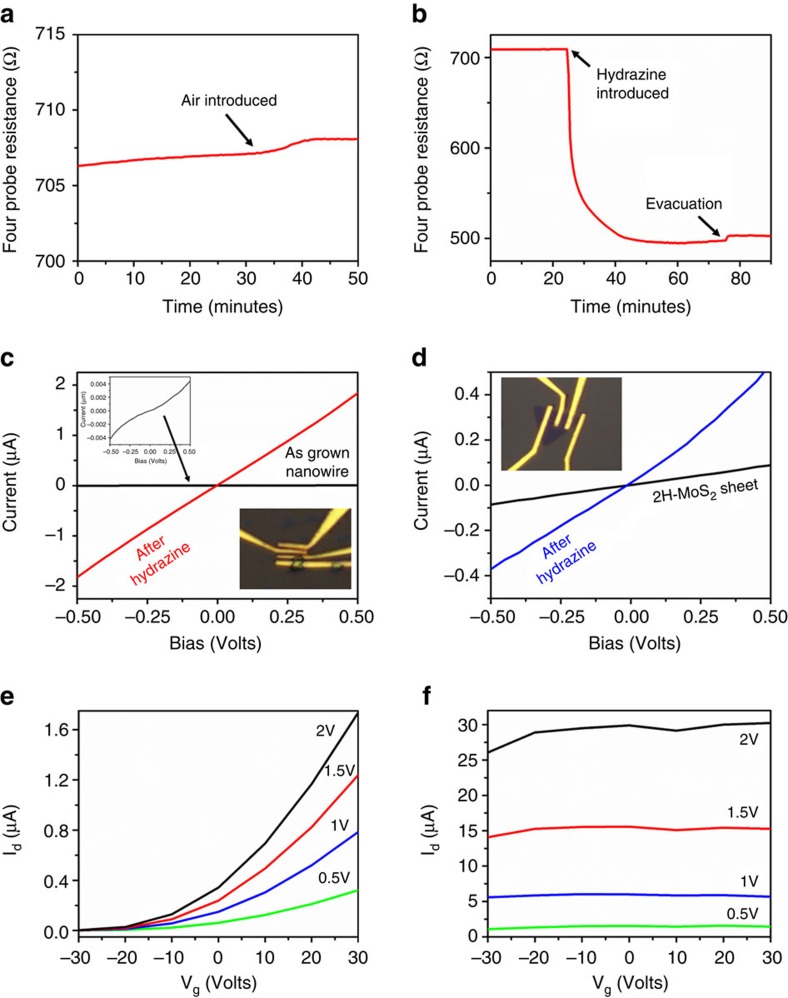
Effects of hydrazine treatment on conductivity of MoS_2_ architectures. (**a**) Four-probe resistance measurement of MoO_x_/MoS_2_ core-shell nanowire array, grown on a non-conductive glass substrate, with exposure to ambient air (shown by arrow) to ∼350 Torr. (**b**) The effects on the four-probe resistance of the MoO_x_/MoS_2_ core-shell nanowire array following the introduction of a small amount (∼15 Torr) of hydrazine (N_2_H_4_) vapour, leading to an almost instantaneous, and irreversible drop in the system resistance. (**c**) Resistance measurement of device fabricated on a small cluster of MoO_x_/MoS_2_ core-shell nanowires (optical micrograph shown in inset). The resistance of the nanowires decreases from ∼133 to 0.3 MΩ following exposure to hydrazine. (**d**) Resistance measurement of device fabricated on single CVD grown 2H-MoS_2_ sheet (optical micrograph shown in inset). The resistance decreases from ∼5.8 to 1.2 MΩ following exposure to hydrazine. (**e**) Drain current-gate voltage analysis of single CVD grown 2H-MoS_2_ sheet and (**f**) following exposure to dilute hydrazine.

Spectroscopic studies indicate that the oxide core is reduced, but do not reveal the effects of hydrazine on the MoS_2_ shell or pure MoS_2_ particles; therefore, *in situ* four-probe resistance and conductivity measurements, as well as gate-dependent measurements, are performed to elucidate the effects of hydrazine on both core-shell nanowires and CVD grown flakes. Initially, the MoO_x_/MoS_2_ core-shell nanowire array is grown directly on a non-conducting glass substrate and mounted on a ceramic holder; two thermocouples and two copper wires (as current leads) are attached to the sample with silver epoxy to measure four point probe resistance prior and following hydrazine vapour exposure. The probe is loaded into a quartz reactor, which was placed inside a tube furnace. The experimental setup is described schematically in the Supplementary Fig. 7. Initial measurements of the resistance at atmospheric pressure and room temperature result in a value of 1.588 kΩ. The system is evacuated to ∼10^−5^ Torr and annealed at 150 °C to remove surface adsorbed moisture; an initial resistance of ∼706 Ω for the as-grown MoO_x_/MoS_2_ core-shell nanowire array is observed. To compare the effect of air and moisture on the resistivity of the core-shell nanowires, the sample is first exposed to ambient air, to a maximum pressure of 350 Torr, which results in a minimal increase in the sample resistance (∼708 Ω), as seen in Fig. 4a. The system is then evacuated again and the resistance stabilized. Then, hydrazine vapours (15 Torr) are introduced to the system. Almost immediately (<30 s), a drop in the sample's resistance is observed, decreasing from ∼710 to ∼495 Ω, which stabilizes after ∼30 min, as shown in Fig. 4b. On evacuation of the system and, therefore, the removal of the hydrazine, the sample's resistance does not significantly increase, maintaining an average resistance of ∼500 Ω. This experimentally confirms that the hydrazine markedly lowers the sample's resistance (increases the conductivity), and thereby, improves the charge transfer characteristics during HER.

For further confirmation, conductivity measurements are performed on a core-shell nanowire back-gated device. The nanostructures are transferred onto Si^++^/SiO_2_ substrates and the electrical contacts (Au) are defined using e-beam lithography (inset of Fig. 4c). Figure 4c shows the change in resistance of a MoO_x_/MoS_2_ core-shell nanowire cluster, before and after hydrazine treatment. It can be clearly observed that after hydrazine treatment (red curve) the current increases sharply in comparison with the untreated device (black curve) for the nanowires. The MoO_x_/MoS_2_ core-shell nanowires show a resistance of ∼133 MΩ before hydrazine treatment, decreasing markedly to ∼0.3 MΩ following the hydrazine treatment.

To isolate the MoS_2_ system from contributions of the reduced oxide core, the resistance measurements are then performed on a single layered CVD grown 2H-MoS_2_ flake^49^. Figure 4d shows the resistance change in the single flake before and after the hydrazine treatment. The MoS_2_ flake shows a resistance of ∼5.8 MΩ before hydrazine treatment; following exposure to hydrazine, the resistance decreases to ∼1.2 MΩ. These results directly support the hypothesis that the hydrazine treatment leads to a decrease in the resistance (or increased conductivity) of MoS_2_. In addition, field-effect gating experiments are performed on a single CVD-grown 2H-MoS_2_ sheet. The effect on drain current (*I*_d_) with changing gate bias (*V*_g_) held at constant drain-source voltages (*V*_ds_) on the untreated MoS_2_ sheet is shown in Fig. 4e. The as-grown MoS_2_ shows a ON/OFF ratio of ∼10^3^ (for *V*_ds_=2 V) and exhibits n-type behaviour, consistent with literature reports for CVD grown MoS_2_ (ref. 50). Following these measurements, this MoS_2_ sheet is treated with hydrazine, thoroughly rinsed with distilled water, and then the field effect gating is measured again (Fig. 4f). In contrast to the untreated device, there is no observable modulation with changing gate voltage and the drain current increases by an order of magnitude. This lack of modulation and increase in current shows the emergence of metallic behaviour following exposure to hydrazine, most likely due to the new states and increased carrier density at the Fermi energy. These experiments support the conclusion that hydrazine electron dopes MoS_2_, in pure sheets as well as the nanowire shell, improving conductivity and electrocatalytic properties.

### Gate-dependent electrochemical HER measurements

**Figure 5:**
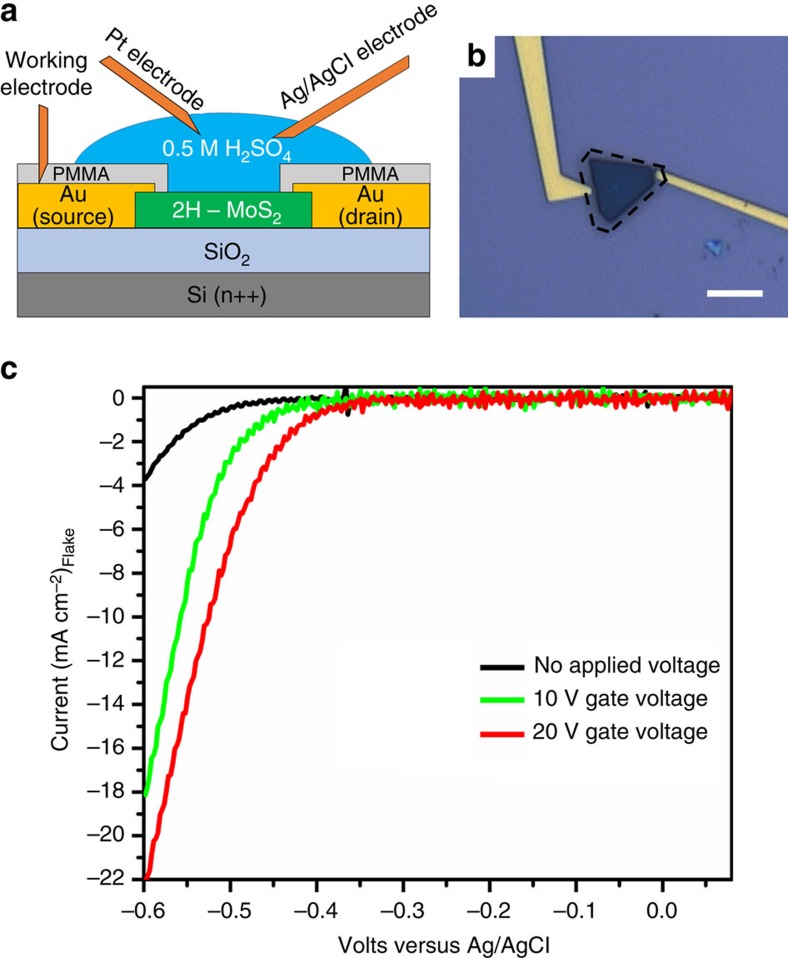
Gate-Dependent HER. (**a**) Schematic of the gate-dependent electrochemical device, with the SiO_2_ layer acting as the gate. (**b**) Optical micrograph of gold contacts and 2H-MoS_2_ single layer flake. The edges of the MoS_2_ flake masked with PMMA are outlined by the black dotted line, the exposed window can be seen as the darker region. Scale bar, 10 μm (**c**) Linear voltammograms from the gate-dependent HER measurements on the single MoS_2_ flake device. The black curve is the activity of the flake with no applied voltage. The green and red curves show the improvement in electrocatalytic activity after applying a positive gate voltage of 10 and 20 V, respectively.

The increase in the conductivity and its correlation to HER catalysis is further corroborated with back gate-dependent electrochemical HER measurements. A single layer 2H-MoS_2_ flake is patterned with gold contacts using e-beam lithography; a schematic representation of the experimental setup is shown in Fig. 5a. The flake and contacts are covered completely with polymethyl-methacrylate (PMMA) polymer and a window (∼140 μm^2^) is opened over to the surface of the flake to allow for electrocatalysis, taking special care to ensure the gold contacts are still covered by the polymer. An optical microscopic image of the device can be seen in Fig. 5b. To test the HER activity, a small drop of 0.5 M H_2_SO_4_ is placed on the flake and linear voltammograms are taken, with a thin platinum wire acting as the counter electrode and AgCl-coated Ag wire acting as the pseudo reference electrode. The results of the gate-dependent HER catalysis measurements are shown in Fig. 5c. At 0 V bias, there is a small, but detectable, HER obtained for the 2H-MoS_2_ flake (black curve). A 10V positive bias is applied to the back gate, inducing a negative charge at the MoS_2_ surface (green curve). The overpotential to drive the HER is reduced (∼500 to ∼ 400 mV versus the Ag/AgCl wire) and the current density increases by over four times. When the gate voltage is increased to 20 V, the overpotential to drive the HER continues to decrease by an additional 50–100 mV and the current increases by five times, when compared with generated current density with no gate bias. This *in situ* observation of the effect of surface electron concentration on HER catalysis directly shows that increasing charge concentration at the MoS_2_ surface can enhance the electrocatalytic activity.

## Discussion

It is essential to understand the interactions between hydrazine and MoS_2_, as well as which mechanism leads to increased conductivity and enhancement in electrocatalysis. Hydrazine has been shown to be an intercalating compound,^51^ can act as a pseudo-reducing agent in TMD systems^52^ and also is a strong reducing agent, which can repair oxidized sulfur sites and inhibit further oxidation of the TMD surface^39^,^53^. Finally, hydrazine has also been shown to be an effective electron dopant in graphene^35^,^36^, carbon nanotubes^37^,^38^, and experiments suggest it can improve the conductivity in PbSe quantum dots^39^,^53^,^54^,^55^,^56^. Due to these numerous possible pathways, it is somewhat challenging to pinpoint the exact mechanism; we investigate these possible mechanisms by identifying key experiments. First, no change in the d-spacing of the MoS_2_ nanowire shell or MoS_2_ sheets is observed following exposure to hydrazine (Supplementary Fig. 8); therefore, hydrazine acting as an intercalating agent is ruled out. Second, hydrazine has been proposed as pseudo-reducing agent^52^ in alkaline conditions. The proposed mechanism of hydrazine forming OH^−^ to act as a pseudo-reduction agent is outlined in Supplementary Note 2. To test this hypothesis, MoS_2_ particles are treated with 0.1 M KOH, but show no improvement in the electrocatalytic characteristics (Supplementary Fig. 9), therefore, the pseudo-reduction of the MoS_2_ surface purely by OH^−^ groups does not adequately explain the mechanism of hydrazine interaction. Third, in the case of MoO_x_/MoS_2_ core-shell nanowires, hydrazine can interact with the oxide core, further reducing the MoO_x_ to make it more conductive^48^. Hydrazine acting as a strong reducing agent is confirmed by CV measurements (Supplementary Fig. 5) as well as XPS studies (Fig. 3).

Finally, electron doping of MoS_2_ by hydrazine should facilitate the adsorption of protons; slight broadening of the sulfur 2p binding energies in the XPS spectra (Fig. 3) is consistent with this modification of the surface energies. Moreover, ultraviolet photoelectron spectroscopy analysis of the hydrazine-treated MoO_x_/MoS_2_ core-shell nanowire system supports this mechanism, providing evidence of electron donation from hydrazine to the conduction band of the MoS_2_ (Supplementary Fig. 10). To characterize the chemical form of hydrazine in the MoO_x_/MoS_2_ core-shell nanowire system, the nitrogen XPS signal (N 1s) is analysed, shown in Supplementary Fig. 11. Following exposure to hydrazine, a N 1s signal arises at ∼400.3 eV, which is not observed in the as-grown sample; the nitrogen signal is in the vicinity of Mo 3p binding energies (analogue to Mo 3d). This nitrogen binding energy corresponds to a surface absorbed amine phase, such as NH_3_ or a sub-amine (NH, NH_2_ and so on)^57^. While XPS makes it difficult to exactly identify the dissociated hydrazine (N_2_H_4_) species, it is clear that amine groups have absorbed on the surface. It has been shown that ammonia, NH_3_, can potentially act as an n-type dopant in metal oxides.^58^,^59^ However, it is more likely that a dissociated radical of hydrazine is acting as the electron donor. Thermal decomposition studies show that N_2_H_4_ readily decomposes to form 2 NH_2_^−^ (ref. 60), a reactive radical species that could contribute electrons to the MoS_2_ surface. Recent theoretical work by Zhang *et al*.^61^ shows that at room temperatures, hydrazine hydrate readily dissociates at a catalyst surface to form radicals, which can donate electrons to the semiconductor surface. The XPS signal of an absorbed amine group supports this theorized mechanism, where dissociated hydrazine radicals (NH_2_* and NH_3_) are present at the MoS_2_ surface and capable of donating electrons. While there are amine groups on the surface, there is no evidence that there is a chemical bonding between the nitrogen and the MoS_2_ shell; the amine group acts as the electron donor. To conclude, in case of core-shell nanowires, hydrazine reduces the oxide core and also acts as an electron dopant for the MoS_2_ shell; in the case of pure MoS_2_ sheets, the change in conductivity is entirely due to electron doping.

**Figure 6:**
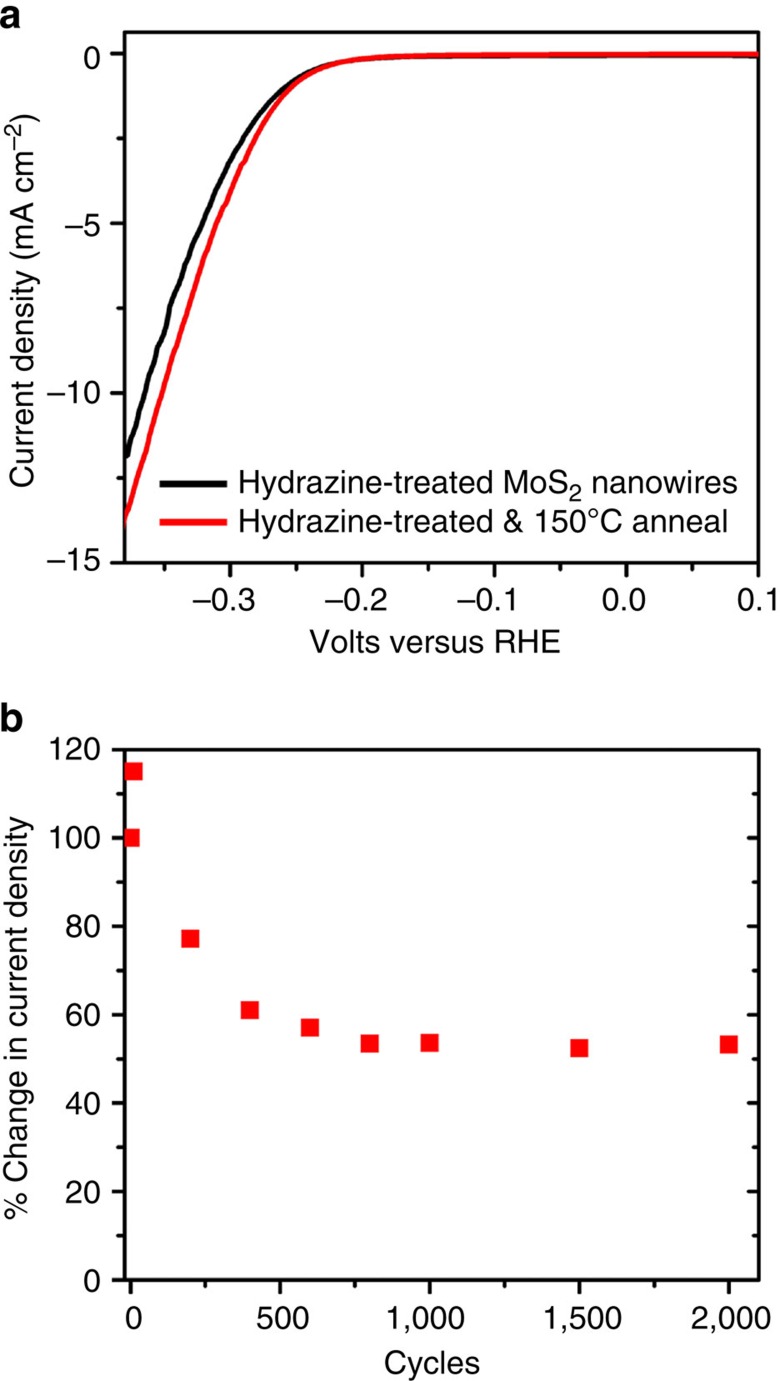
Stability studies of hydrazine-treated MoO_x_/MoS_2_ core-shell nanowire. (**a**) Linear voltammetry for MoO_x_/MoS_2_ core-shell nanowires treated with Hydrazine (red curve), then annealed at 150 °C for 1 h (red dashed line). (**b**) Stability of hydrazine-treated MoO_x_/MoS_2_ core-shell nanowires over 2,000 cycles. Current densities are normalized to ∼24 mA cm^−2^ as 100%.

Finally, for commercialization and technologically viable use of TMDs for hydrogen production, thermal stability and long-term durability are required. Chemically exfoliated 1T-MoS_2_ sheets are sensitive to temperature as the metastable phase transformation is reversed on annealing (loss of catalytic activity)^21^,^31^. Electrocatalytic activity of hydrazine-treated nanowires before and after annealing at 150 °C for 1 h under argon are shown in Fig. 6a. There is no significant change in HER onset potential or current density, which demonstrates that the effect of the hydrazine treatment is not merely due to physisorption. The electrochemical durability at room temperature of the hydrazine-treated nanowires is also investigated, shown in Fig. 6b. The initial current density at 0.35 V versus RHE is ∼24 mA cm^−2^ following the hydrazine treatment, which is set as 100%. After 10 scans, the current density at 0.35 V actually increases, which has been seen in other reports^29^, but slowly decreases to ∼60% of its initial activity, stabilizing after around 400 cycles to an average of ∼13 mA cm^−2^. This degradation may be due, in part, to decomposition of the oxide core, since the MoS_2_ shell is thinner than in other reports^29^.

In conclusion, exposure of MoO_x_/MoS_2_ core-shell nanowires to an aqueous hydrazine solution leads to a marked improvement in electrocatalytic activity. We observe a 100 mV improvement in the HER onset potential (from approximately −200 mV to approximately −100 mV versus RHE) and also an exponential increase in generated current density (from 2 to 22 mA cm^−2^at −0.35 V versus RHE). Furthermore, the TOF for the core-shell nanowires increases five-fold following the hydrazine treatment, from 0.04 to 0.2 s^−1^, due to the synergistic reduction of the oxide core and the electron doping of the MoS_2_ shell. In the case of MoS_2_ bulk powder, the TOF increases by nearly twofold, from 0.03 to 0.05 s^−1^, since electron doping is the only contributing factor. Surface characterization studies reveal that the change in catalytic properties does not result from a phase transformation, but is due to enhanced conductivity. The increased conductivity, as result of hydrazine treatment, is shown in MoO_x_/MoS_2_ core-shell nanowires, 2H-MoS_2_ particles, and single layer sheets, by utilizing field effect gating experiments, conductivity, and spectroscopic techniques. Hydrazine is shown to act as an electron dopant; dissociated amine radicals donate electrons to the MoS_2_ surface, facilitating electrocatalysis. The reported hydrazine modifications can be performed in ambient conditions and on the order of minutes, when compared to conventional techniques. This is one of the first known investigations into the effect of hydrazine exposure in 2D layered chalcogenides for electrocatalysis application. Understanding the effects of hydrazine on TMDs in catalysis can lead to a fundamental breakthrough in the areas of electrochemistry and material science.

## Methods

### Electrochemical testing measurements

To examine the electronic properties of the nanowires, 1% by volume hydrazine (N_2_H_4_) and water solution (20 μl) was dropped directly on the nanowire array and allowed to dry in air (∼10 min). Electrodes were prepared by gently dispersing the hydrazine-treated nanowire array in distilled water, then transferred to 3 mm diameter glassy carbon tip electrodes (∼70 μg cm^−2^). A thin layer of 5% Nafion (∼2.7 μl cm^−2^) was further applied on the electrodes to prevent delamination of the active material into solution, but still allowing for proton transport. The electrochemical analysis was performed by cyclic voltammetry in 0.5 M H_2_SO_4_ solution (pH=0) using a graphite rod as counter electrode and Ag/AgCl as a reference electrode (+0.210 V versus RHE). Nitrogen was bubbled vigorously through the electrolyte to remove any oxygen from solution. The sample was cycled multiple times to remove surface contamination and ensure steady state conditions.

For electrochemical measurements of bulk MoS_2_ powder, the powder was dispersed by sonication in distilled water (100 mg MoS_2_ per 10 ml H_2_O) and drop-cast onto glassy carbon electrodes (∼1 mg cm^−2^). For the hydrazine treatment, the dried MoS_2_ dispersion on glassy carbon was drop-cast with 1% hydrazine solution and allowed to dry, then coated with Nafion, following the exact methods as with the core-shell nanowires.

### Gate device fabrication and conductivity measurements

For fabrication of the MoS_2_ sheet devices, MoS_2_ monolayer sheets were grown using standard CVD procedure^49^ and transferred onto 300 nm SiO_2_/Si^++^ substrates by the PMMA assisted transfer method. PMMA layers were then washed by acetone under 50 °C followed by IPA rinse. In the case of the MoO_x_/MoS_2_ core-shell nanowire device, the nanowires are first dispersed by sonication in water, then directly drop-casted onto the 300 nm SiO_2_/Si^++^ substrates. PMMA was spin-coated at 3,000 r.p.m. for 60 s, followed by a soft bake at 180 °C. The electrode patterns were defined by using standard e-beam lithography method (5/40 nm Ti/Au contacts). This was followed by a lift-off process to achieve the final device configuration. Initial conductivity measurements and gate-dependent measurements were performed on these devices in vacuum. To test the effect of hydrazine, another e-beam lithography step was performed to selectively expose a region within the device channel to isolate the contacts. Hydrazine treatment was performed by placing a 1% dilute hydrazine solution for 5 min to a few hours. The devices are then thoroughly rinsed with distilled water and electrical measurements performed again in vacuum.

### Characterization instrumentation

FEI Tecnai F20 TEM was used for high-resolution TEM. SEM imaging utilizes a FEI Quanta FEG 400 Scanning Electron Microscope. XPS analysis is performed using a Physical Electronics 5600ci XPS system with an Al Kα radiation source. All XPS spectra are calibrated by the position of the C 1s peak. The carbon signal used for calibration results from surface absorbed hydrocarbons, which have a characteristic peak location of 284.5 eV. The Raman spectroscopic analysis of the nanowire arrays is performed using a Renishaw Invia Micro Raman system with a 633 nm HeNe laser. Raman system is calibrated using single crystal Si wafer, with characteristic peak at 520.0 cm^−1^.

### Data availability

The data that support the findings of this study are available on request from the corresponding author G.G.

## Additional information

**How to cite this article:** Cummins, D. R. *et al*. Efficient hydrogen evolution in transition metal dichalcogenides via a simple one-step hydrazine reaction. *Nat. Commun.* 7:11857 doi: 10.1038/ncomms11857 (2016).

## Supplementary Material

Supplementary Information Supplementary Figures 1-11, Supplementary Notes 1-2 and Supplementary References

## Figures and Tables

**Table 1:** Electrochemical parameters with hydrazine treatment of MoS_2_.

	**HER onset (Volts versus RHE**)	**Current density at -0.4 V (mA cm**^−2^**)**	**Tafel slope (mV per decade)**	**Exchange current density** ***i***_***0***_ **(A cm**^−2^**)**	**No of sites per cm**^2^	**Turnover frequency (s**^−1^**)**_**η=150 mV,200 mV**_
As-grown MoO_x_/MoS_2_ Nanowires	−0.200	2.0	90	7.5 × 10^−6^	5.9 × 10^14^	0.04
Hydrazine-treated MoO_x_/MoS_2_ Nanowires	−0.100	22.0	50	4.5 × 10^−5^	6.0 × 10^14^	0.2
Bulk MoS_2_ particles	−0.250	0.75	134	8.4 × 10^−6^	9.0 × 10^14^	0.03
Hydrazine-treated MoS_2_ particles	−0.200	2.0	108	1.7 × 10^−5^	9.8 × 10^14^	0.05
