# Supplementary material for: Efficient hydrogen evolution in transition metal dichalcogenides via a simple one-step hydrazine reaction
Source: Nat Commun. 2016 Jun 10;7:11857. doi: 10.1038/ncomms11857 (PMC4906413; doi:10.1038/ncomms11857)
Supplement: Supplementary Information — Supplementary Figures 1-11, Supplementary Notes 1-2 and Supplementary References [file ncomms11857-s1.pdf]

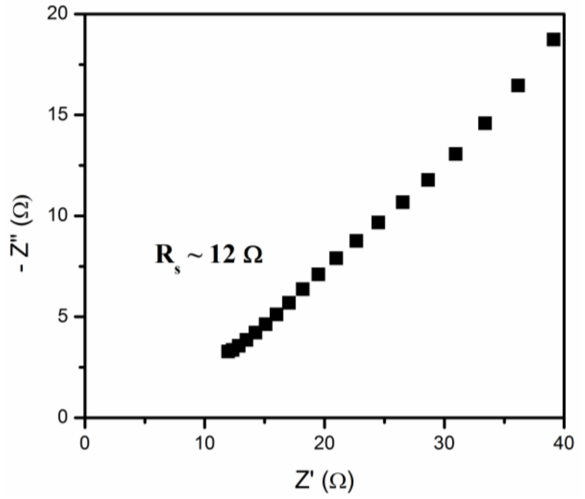

Supplementary Figure 1. Impedance Spectrum for iR Correction. Nyquist plot showing the impedance of hydrazine treated MoO_x/MoS_2 core-shell nanowires dispersed on glassy carbon electrode with graphite counter, resulting in a system resistance of ~12 ohms. Used for iR correction of electrochemical data.

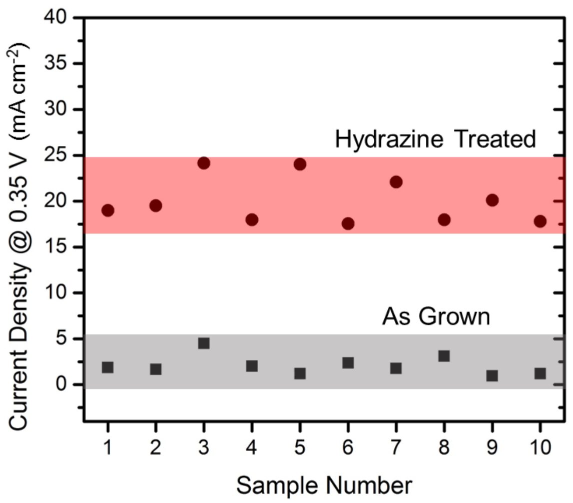

Supplementary Figure 2. HER Electrocatalysis Reproducibility Study. Reproducibility of the hydrazine treatment. Ten separate MoO_x/MoS_2 core-shell nanowire arrays were treated with 1% aqueous N_2H_4 and electrochemically tested, with similar catalyst loading. The current

density at -0.35 V vs. RHE averages 19.6 mA cm^{-2} and is consistently improved over the as grown core-shell nanowire activity.

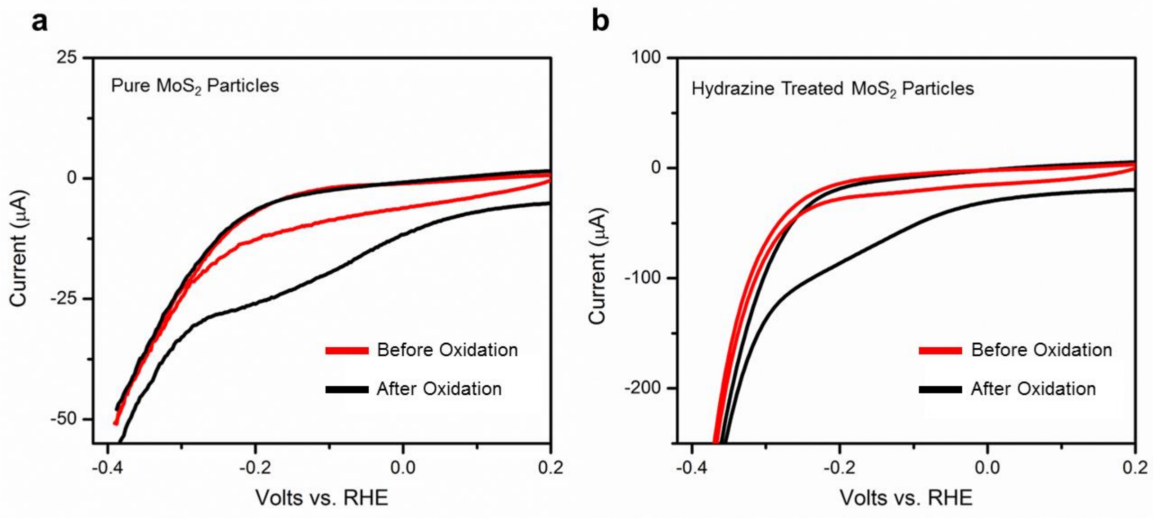

Supplementary Figure 3. Oxidation of MoS_2 Particles for Surface Area Calculation. Cyclic voltammograms for MoS_2 particles showing the Mo^{3+} to Mo^0 region (-0.3 - 0.0 V) used for calculation of number of MoS_2 sites. Curves in red are obtained before potential is driven to high oxidizing potentials, while black curves show the oxidized MoS_2 particles.

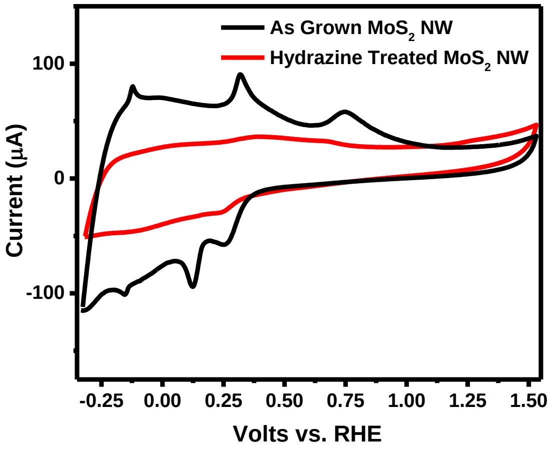

Supplementary Figure 4. Oxidation of MoO_x/MoS_2 Particles for Surface Area Calculation Cyclic voltammograms for MoO_x/MoS_2 core-shell nanowires, both as grown (black) and after hydrazine treatment (red), following HER measurements. As grown nanowires show a

convoluted -0.3 - 0.5 V region due to the combination of core and shell oxidation-reduction peaks, while hydrazine treated nanowires seem feature less due to the reduction of the MoO_3 core.

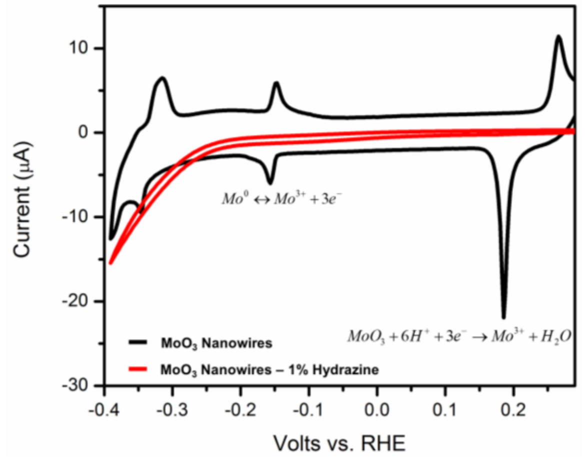

Supplementary Figure 5. Effects of Hydrazine on MoO_3 Nanowires. Cyclic voltammograms for pure MoO_3 nanowires, both as grown (black) and after hydrazine treatment (red).

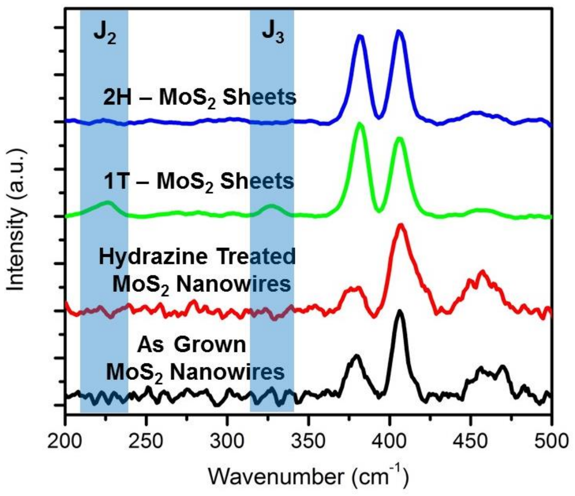

Supplementary Figure 6. Raman Analysis. Raman spectroscopy of MoO_x/MoS_2 core-shell nanowire arrays as grown (black curve) and after hydrazine treatment (red curve). Chemically

exfoliated 1T-MoS_2 sheets (green curve) and 2H-MoS_2 sheets (blue curve) published by Cummins, et al.[1] are shown for comparison. The locations of the J_2 and J_3 for 1T-MoS_2 is shown by the shaded area. The Raman spectrum for the as-grown nanowires shows that these nanowires are 2H-phase and there is no evidence of a phase transition after the addition of hydrazine.

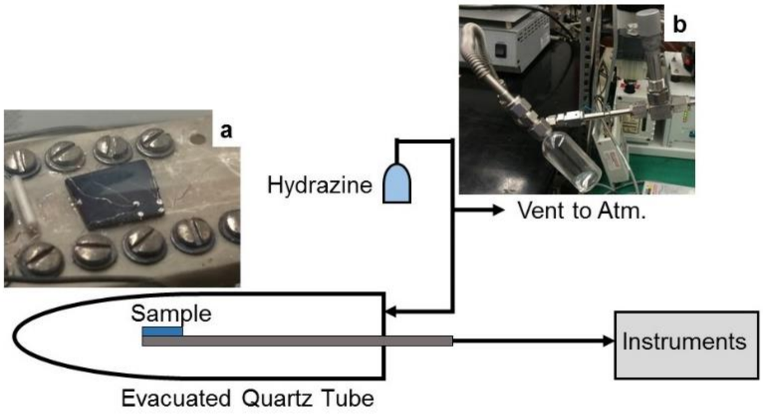

Supplementary Figure 7. *In Situ* Conductivity Experimental Set up. Schematic showing *in situ* conductivity experimental set up. **(a)** Photograph detailing the four point probe connection to the MoS_2 nanowire array grown on glass. **(b)** Photograph showing a small vial with a small amount of liquid hydrazine at the inlet to the evacuated quartz tube. When the system is under vacuum, only the saturated vapor is allowed into the system.

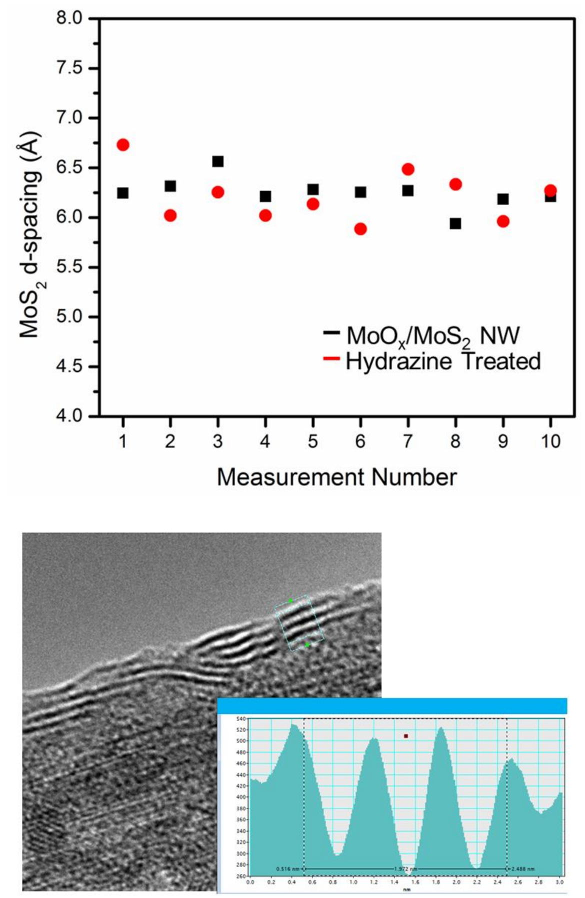

Supplementary Figure 8. MoO_x/MoS_2 Core-Shell Nanowire d-spacings Distribution of d-spacing of MoS_2 shell of MoO_x/MoS_2 core-shell nanowires as grown (black) and following hydrazine treatment (red). An example of HRTEM of the as grown MoO_x/MoS_2 core-shell nanowire is shown along with the line scan to measure d-spacing. Careful measurement of the interlayer spacing of the MoS_2 shell shows no statistically significant change in the interlayer spacing of the MoS_2 shell, with an average d-spacing of ~6.23 +/- 0.21 Å, which is typical for 2H-MoS_2.

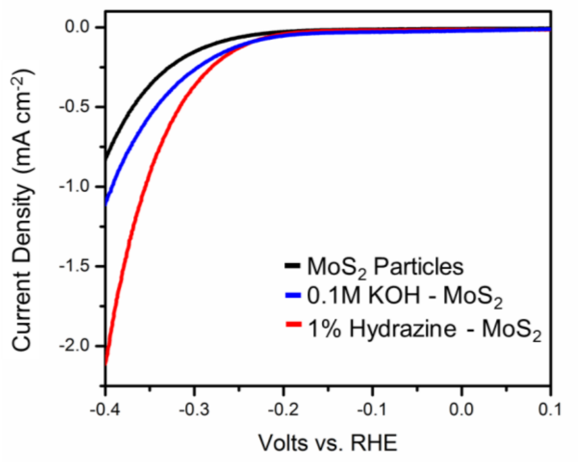

Supplementary Figure 9. MoS_2 Particles and KOH. Linear voltammograms of pure MoS_2 particles before (black curve) and following exposure to 0.1M KOH (blue curve), and exposure to dilute hydrazine (red curve) for comparison.

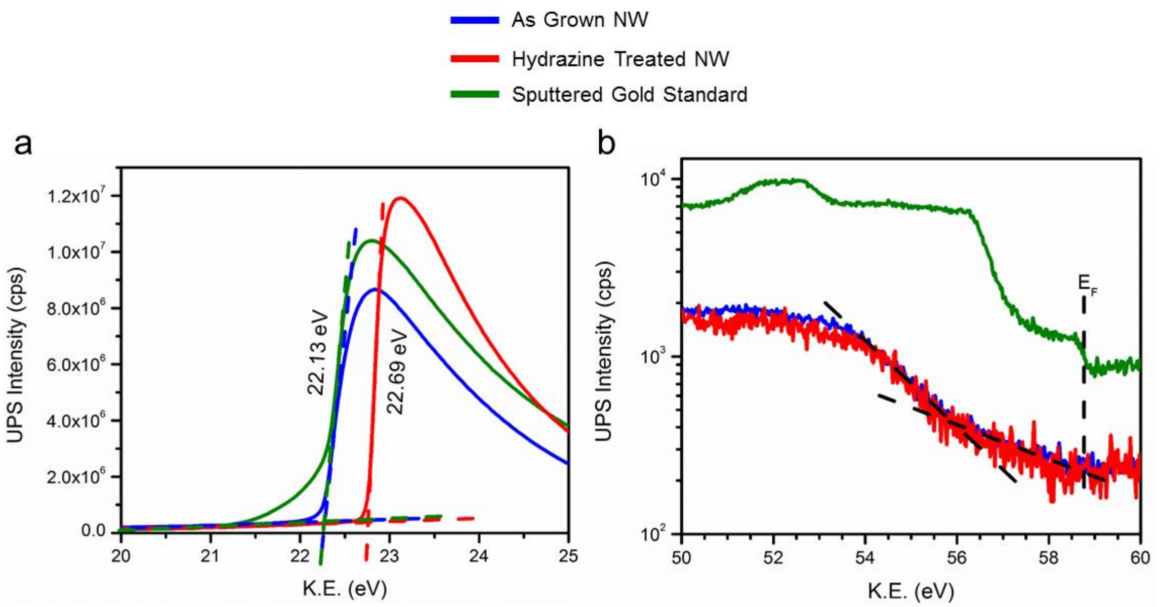

Supplementary Figure 10. Ultraviolet Photoelectron Spectroscopy (UPS). UPS He II (40.8 eV) radiation spectra of MoO_x/MoS_2 core-shell nanowire arrays as grown (blue) and following treatment with 1% hydrazine (red). Gold is sputtered onto a portion of the sample in order to act as a standard for determination of the Fermi level (green). a) Detail showing on-set edge of secondary photoelectrons. This shows the shift in on-set (*i.e.* work function of the MoO_x/MoS_2

core-shell nanowire array) by ~0.5 eV following exposure to dilute hydrazine. b) Detail showing the Fermi energy of the gold sputtered standard. Since the gold is in ohmic contact with the MoO_x/MoS_2 core-shell nanowire array, it can be assumed that the Fermi level of the MoO_x/MoS_2 core-shell nanowires is equal to that of the gold standard. Dotted black lines show states in the valence band of the MoS_2.

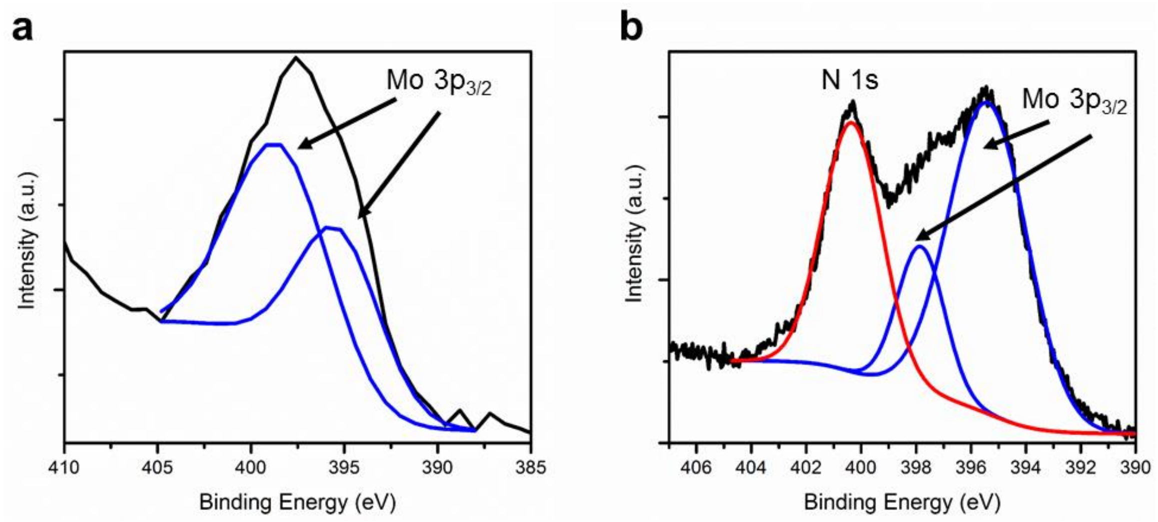

Supplementary Figure 11. XPS of Nitrogen Species in Hydrazine Treated MoO_x/MoS_2 Core-Shell Nanowires a) XPS spectrum of as grown MoO_x/MoS_2 core-shell nanowires. The Mo $3p_{3/2}$ peaks (blue curves) correspond to the primary Mo 3d binding energies, as shown in Manuscript Figure 3. b) XPS spectrum of MoO_x/MoS_2 core-shell nanowires following hydrazine treatment. Nitrogen, N 1s, signal (red curve) at ~400.3 eV is indicative of a surface bound amine phase (NH_3, NH_2^-, etc.)

Supplementary Note 1. Raman Spectroscopy

Raman spectroscopy is used to determine the crystal phase of the MoS_2 shell, which is trigonal prismatic (2H) as synthesized. Bulk 2H-MoS_2 has Raman excitations at 382 and 407

cm^{-1}, which corresponds to E^1_{2g} and A_{1g} phonons. Second order phonons appear as a broad peak at ~453 cm^{-1}, denoted as 2LA(M).[2,3] It has been shown that 2 unique peaks arise at 230 and 350 cm^{-1}, denoted at J_2 and J_3, when the phase transition to the metal stable orthorhombic 1T-MoS_2 occurs.[4] This 1T-MoS_2 spectrum is shown at the green curve for comparison.[1]

Supplementary Note 2. Pseudo-Reduction Mechanism by OH^-

It has been proposed that a strongly basic solution can act as a pseudo-reducing agent in semiconductors. When dispersed in water, hydrazine dissociates to form a basic solution:

$$N_2H_4 + H_2O \rightarrow [N_2H_5]^+ + OH^- \quad (1)$$

It has been shown that in aqueous basic solutions, chalcogenide semiconductors can be hydrolyzed, which pseudo-reduces the semiconductor at the surface.[5] The proposed reaction mechanism for the pseudo-reduction of the MoS_2 layers is shown[6]:

$$xOH^- + MoS_2 \rightarrow MoS_{2-x}O_x + S^{2-} + xH^+ \quad (2)$$

$$xS^{2-} \rightarrow \frac{1}{x}S_x + 2e^- \quad (3)$$

$$0.3e^- + MoS_{2-x}O_x \rightarrow [MoS_{2-x}O_x]^{0.3-} \quad (4)$$

Following exposure to dilute KOH, there is a very slight increase in the generated current density of 2H-MoS_2 particles, but almost no effect on HER overpotential. It is clear that pseudo-reduction is not a viable theory for interaction between hydrazine (and formed OH^- groups) and the MoS_2 surface.

Supplementary References

1. Cummins, D. R. *et al.* Catalytic Activity in Lithium-Treated Core–Shell MoOx/MoS2 Nanowires. *The Journal of Physical Chemistry C* **119**, 22908-22914 (2015).
2. Li, H. *et al.* From Bulk to Monolayer MoS2: Evolution of Raman Scattering. *Adv. Funct. Mater.* **22**, 1385-1390 (2012).
3. Frey, G. L. *et al.* Raman and Resonance Raman Investigation of MoS2 Nanoparticles. *Phys. Rev. B* **60**, 2883-2892 (1999).
4. Eda, G. *et al.* Photoluminescence from Chemically Exfoliated MoS2. *Nano Lett.* **11**, 5111-5116 (2011).
5. Subba Rao, G. V. S., M.W. *Intercalation in Layered Transition Metal Dichalcogenides*. 99-199 (D. Reidel Publishing Company, 1979).
6. Schollhorn, R., Sick, E. & Lerf, A. Reversible Topotacti Redox Reactions of Layered Dichalcogenides. *Mater. Res. Bull.* **10**, 1005-1012 (1975).
